# A Case of Infantile Perianal Pyramidal Protrusion Masquerading As Imperforate Anus at Birth

**DOI:** 10.7759/cureus.18491

**Published:** 2021-10-05

**Authors:** Mohammad Adnan, Deepika Sankaran, Janardhan Mydam, Prashant Malviya, Imteyaz Khan

**Affiliations:** 1 Neonatology, Indiana University Health Ball Memorial Hospital, Muncie, USA; 2 Division of Neonatology, Department of Pediatrics, University of California Davis, Sacramento, USA; 3 Neonatology, John H. Stroger, Jr. Hospital of Cook County, Chicago, USA; 4 Neonatology, Saint Peter’s University Hospital, New Brunswick, USA

**Keywords:** infantile perianal pyramidal protrusion, anal skin tag, neonate, imperforate anus, rectal mass, perianal protrusion

## Abstract

Infantile perianal pyramidal protrusion (IPPP) is an uncommon and underreported benign cutaneous lesion characterized by a protrusion from the anal orifice. It is also believed to be often mistaken for other conditions. The unawareness of this lesion may be responsible for underreporting and an excessive concern both in providers and in parents. Timely diagnosis and reassurance need to be emphasized in the provider community.

We report an interesting case of IPPP on the first day of life, which was erroneously diagnosed as imperforate anus at her delivery.

## Introduction

The term infantile perianal pyramidal protrusion (IPPP) was first coined by Kayashima et al. in 1996 when they reported it for the first time [1]. Since then, close to 110 cases have been reported in the literature [2]. This benign condition is not believed to be rare but is often erroneously interpreted as a vascular malformation, skin tags, or sometimes even a result of sexual abuse in older children [3]. Moreover, it has been only reported a few times in neonates. IPPP is predominantly diagnosed in females and is marked by a pyramidal-shaped fleshy pink protrusion located on the midline raphe of the perineum, usually anterior to the anus [2,4]. Here, we report a case of IPPP in a one-day-old infant which had completely regressed by 3 days of life.

## Case presentation

**Figure 1:**
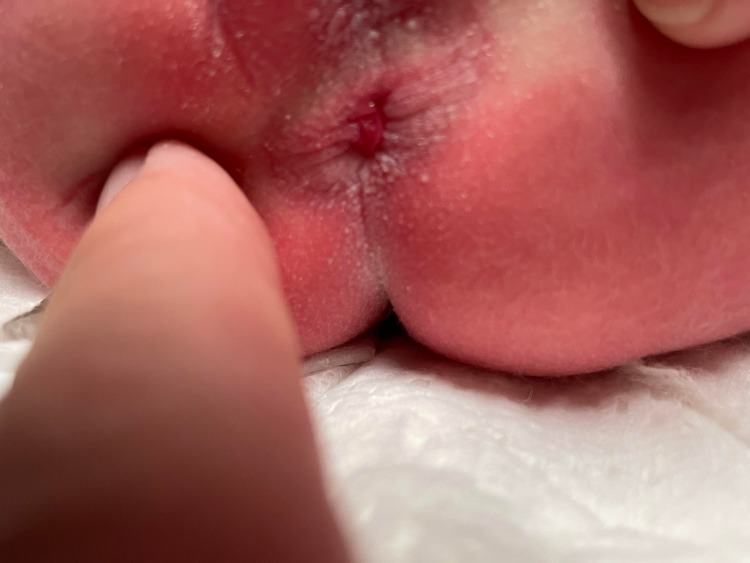
Picture taken at 6 hours of life showing pyramidal-shaped protrusion completely covering anal orifice

A full-term Caucasian female infant was delivered at 39 weeks and 1 day gestational age via normal vaginal delivery to a 28-year-old Gravida 2 now Para 2 mother. No significant maternal history except for basal metabolic index (BMI) > 30 and diet-controlled gestational diabetes was reported. Her birth weight was 2790 grams (16th percentile) and Apgar scores were 8 and 9 at 1 and 5 minutes, respectively. The infant did not require any resuscitation at birth. During the initial assessment by her nurse, the absence of an anal orifice was noted. The on-call neonatal provider evaluated the baby and diagnosed her with imperforate anus due to the absence of an anal orifice. The infant was made nil per oral and started on intravenous fluids. She was subsequently examined by the neonatologist. On examination, a fleshy pink pyramidal mass of approximately 4 mm × 2 mm was seen covering the anal orifice (Figure 1). The rest of the abdominal exam was described as soft non-distended and nontender with normal bowel sound without any organomegaly. A lubricated 5 Fr catheter was passed into the rectum with ease on gentle manipulation. Following this, the baby passed meconium 6 hours after birth. No other anomalies were noted on a detailed physical examination. The baseline abdominal radiograph was also within normal limits. Oral feedings were resumed, and the infant continued to have regular bowel movements. Detailed family history revealed a history of intestinal polyps in the family. No history of similar perianal lesions in any family members was reported. The family was reassured and explained the benign nature of the lesion.

A decision was made to hold off on pediatric surgery consult at this time. The mass became less prominent and gradually disappeared by day 3 of life. The infant was otherwise doing well and was discharged home in stable condition. During outpatient follow-up with the pediatrician, no recurrence of the IPPP was noted until a 4-week follow-up. However, the baby was switched to elemental formula because of suspected cow’s milk protein allergy based on a history of blood-stained stools. Mother had opted for formula feeding so exclusive breast milk could not be offered as a treatment. However, following this change in infant formula, no further bloody stools were reported further. The infant continued to grow well with a normal stooling pattern.

## Discussion

IPPP also referred to as infantile perianal protrusion or infantile perineal protrusion was originally described as a skin tag. After its first description by Kayashima et al in 1996, it has been only reported in case reports, case series, and observational studies [1-6]. It has been described in various shapes and sizes ranging from pyramidal (most common) to papular, peanut-shaped, leaf-like, tongue-like, and hen’s crest-shaped. IPPP is usually located posterior or concomitantly anterior and posterior to the anus with colors ranging from flesh-colored to red or pale [4-8]. Its location and color may vary based on the duration and condition of the perineal skin. The differential diagnosis includes, but is not limited to, rectal prolapse, rectal polyp, external hemorrhoids, sentinel tag of anal fissure, hemangiomas and other vascular malformations, granulomatous perineal lesions of Crohn’s disease, sexual abuse, condyloma, molluscum contagiosum, and skin tags. Moreover, if this lesion is present in a neonate covering the anal orifice, it can easily be confused with imperforate anus. This may lead to discontinuation of feeds, unnecessary workup (including radiographs and contrast studies), and even transfer to a tertiary hospital for pediatric surgery evaluation.

The pathogenesis of IPPP is unknown, but most experts opine that the lesion is caused by one of the following mechanisms. First, a congenital form of IPPP may result from weakness of the median raphe. Alternately, it may be a remnant of the urogenital septum as evidenced by reports of new lesions developing after episodes of constipation [9,10]. This type may be inherited, occurs in many members of the family, and is often seen in males. A perineal nodule may be a developmental anomaly that occurs in females that gradually involutes with age. Embryologically, the perineum is formed by elongation of the urogenital septum during the growth of the fetus. Therefore, the perineal nodule may be a remnant of the projected tip of the urogenital septum [3-5]. The second type is an acquired form given its association with chronic diarrhea, perianal fistulas, and anal fissures speculated to be secondary to mechanical irritation and friction from wiping [2,3]. Finally, the third type is attributed to a rearrangement of fibrous tissue caused by inflammation related to genital lichen sclerosus et atrophicus (LSA) [7,8]. Histology findings in IPPP are usually nonspecific and not required for diagnosis. Histopathologic examinations reported in the literature have demonstrated: epidermal acanthosis, slight thickening and/or elongation of the rete ridges, dilatation of capillary blood vessels, and dilatation of lymphatics along with local infiltration by lymphocytes and eosinophils in the dermis (especially upper), or an almost normal picture [4,6].

Diagnosis is usually based on history and a careful physical exam. In doubtful cases, ultrasonography (US) and dermoscopy may aid in timely diagnosis and thus avoid unnecessary biopsy and histology [11,12]. US remains the most useful imaging modality for pediatric genital lesions. Addition of color Doppler imaging allows for quick identification of normal blood vessels and helps to differentiate abnormal vascular structures [13,14]. The presence of perianal lesions can create significant anxiety for parents and is prone to misdiagnosis. However, many studies and case reports including ours noted a spontaneous resolution of lesions over time. Our case being among only a few reported in the immediate newborn period with resolution within 3 days is an important observation to understand the natural history of this lesion. However, if it is suspected to be of the acquired form, management of the precipitating factor is warranted, including dietary changes and aggressive treatment of constipation or diarrhea when present. If there is clinical or pathologic evidence of LSA, then treatment of the underlying condition should be initiated with topical corticosteroids [15].

## Conclusions

In conclusion, IPPP may not be as rare as it is reported in the literature, and awareness of this condition is paramount in avoiding unnecessary workup. Diagnosis can be easily made on history and a careful physical exam. Most lesions would resolve spontaneously or by treating the underlying cause. However, more studies are needed to document the natural course of this benign condition.
